# Prognostic significance of Human epidermal growth factor receptor-2 expression in patients with resectable gastric adenocarcinoma

**DOI:** 10.1186/s12957-019-1652-2

**Published:** 2019-07-11

**Authors:** Hyeongbin Kim, SangHyuk Seo, KwangHee Kim, Yo-Han Park, MinSung An, HyungJoo Baik, ChangSoo Choi, SangHoon Oh

**Affiliations:** Department of Surgery, Busan Paik Hospital, Inje University, Bokgiro 75, Busanjin-gu, Busan, Republic of Korea

**Keywords:** Gastric adenocarcinoma, Human epidermal growth factor receptor 2, Five-year survival rate, Prognostic factors

## Abstract

**Background:**

The purpose of this study was to investigate the correlation between human epidermal growth factor receptor 2 (HER2) overexpression and clinicopathologic factors and overall survival rate in patients who underwent curative gastrectomy for gastric adenocarcinoma.

**Methods:**

Among patients who underwent curative gastrectomy for gastric adenocarcinoma at Inje University Paik Hospital from January 2012 to December 2015, 782 patients underwent an immunohistochemical analysis to evaluate HER2 expression levels. Clinicopathologic records that were collected from a gastric cancer database were retrospectively reviewed to identify clinicopathologic factors and survival rates of the patients.

**Results:**

HER2 overexpression was detected in 166 patients (21.2%). There was a statistically significant correlation between HER2 expression level and sex (*p* = 0.013), histologic differentiation (*p* < 0.001), Lauren classification (*p* < 0.001), and T pathologic stage (*p* = 0.022). There were no statistically significant relationships between HER2 expression level and overall 5-year survival rate (*p* = 0.775) and overall 5-year survival rate of gastric adenocarcinoma classified according to the TNM stage (stage I: *p* = 0.756, stage II: *p* = 0.571, stage III: *p* = 0.704). The HER2 expression level was not affected by the overall 5-year survival rate in the uni- and multivariate analyses.

**Conclusions:**

In this study, the HER2 overexpression rate in gastric adenocarcinoma was 21.2% and was observed in well- and moderately differentiated types according to histologic differentiation, intestinal type according to the Lauren classification, male, and low T stage. There was no correlation between HER2 expression level and overall 5-year survival rate, and HER2 expression level was not associated with independent prognostic factors.

## Introduction

In Korea, the detection rate of early gastric cancer has increased from 24.8 to 57.6% over the past 20 years due to the implementation of national checkups and the development of diagnostic techniques [1]. Furthermore, the overall 5-year survival rate of gastric cancer has increased from 42.8 to 74.4% due to the standardization of gastrectomy, increased operator expertise, and development of surgical tools [2]. However, the mortality rate due to gastric cancer remains high (16.7 per 100,000 patients) [3] because not all gastric cancers are diagnosed at early stages and various genetic mutations are present in gastric cancer, leading to various anti-cancer treatment responses. The overexpression or abnormal expression of tyrosine kinase receptors plays crucial roles in the survival and proliferation of gastric cancer cells. Based on this observation, receptor ligands, receptors, tyrosine kinase domains in the lower portions of the receptors, and signaling molecules have been targeted for treating cancer [4].

Human epidermal growth factor receptor 2 (HER2), a target used in breast cancer treatment, is the second member of the epidermal growth factor receptor (EGFR) family and is a type of receptor tyrosine kinase. It is a 185-kD glycoprotein that was discovered when the *NEU* oncogene was studied in rats. HER2 is encoded by the *ERBB* gene that is located on human chromosome 17, q21 [5]. HER2 is a prognostic factor for invasive breast cancer and shows gene amplifications in approximately 20% of breast cancers, resulting in its overexpression and providing a treatment target [6]. In addition to its significance in breast cancer, *HER2* gene amplification and protein overexpression have been observed in esophageal, gastric, and colon cancers, and *HER2* plays an important role in cancer cell proliferation, apoptosis, adhesion, angiogenesis, and aggressiveness [5].

The ToGA study [7] used trastuzumab, an agent used to treat HER2-positive breast cancer, as a treatment agent for unresectable gastric cancer. Patients with HER2-positive gastric cancer or gastroesophageal cancer who were treated with trastuzumab (trastuzumab, cisplatin, and fluoropyrimidine [oral capecitabine or IV fluorouracil]) have shown better survival rates than those who were not treated with trastuzumab. HER2 overexpression has been investigated in many studies and has been reported to appear with frequencies of 6–30% in gastric cancer [8–22]. Clinicopathologic factors that are associated with HER2 overexpression have also been reported to be highly expressed in well-differentiated adenocarcinomas and intestinal-type adenocarcinomas according to Lauren classification; however, some studies have shown differences [12–23]. Additionally, there have been various reports on HER2 overexpression, some of which have demonstrated that the survival rate of patients with gastric cancer and HER2 overexpression are not related, while others have found that HER2 overexpression is an independent prognostic factor in gastric cancer [10, 12–22].

The purpose of this study was to investigate HER2 overexpression by immunohistochemical (IHC) analysis in gastric adenocarcinoma tissues that were obtained by curative gastrectomy. In addition, we compared clinicopathologic factors and survival rates with HER2 expression level to identify factors associated with HER2 overexpression. Furthermore, HER2 overexpression was examined to determine its value as an independent prognostic factor.

## Study method

### Study participants

Among 810 patients who underwent curative gastrectomy to treat gastric adenocarcinoma at Inje University Paik Hospital, from January 2012 to December 2015, this study included 782 patients after excluding 28 patients. Twenty-eight patients who were excluded from our study underwent curative gastrectomy after endoscopic submucosal dissection and did not undergo IHC analysis because of the absence of residual lesion in the pathologic result. Fresh gastric adenocarcinoma tissue obtained during curative gastrectomy was created simultaneously with slides for the pathological stage as well as slides for IHC analysis. The clinicopathologic records were collected from a gastric cancer database and were analyzed retrospectively to identify pathologic features of the patients. The pathologic stage was defined by the 7th edition of the TNM classification system from the American Joint Committee on Cancer [24]. Patients with TNM stage Ib were treated with oral 5-fluorouracil, and patients with TNM stages II and III were treated with S-1 [25] and xelox (intravenous oxaliplatin and oral capecitabine) [26] as adjuvant chemotherapy.

This study was approved by the Institutional Review Board of the Inje University Paik Hospital (IRB No. 17-0104).

### IHC methods

After the curative gastrectomy, gastric adenocarcinoma tissues were sent to the department of pathology to identify the gastric adenocarcinoma pathologic stage. In brief, 4-μm-thick samples were cut and slices were prepared on glass slides, to which aminoprophyltriethoxysilane (APEAS, Sigma, USA) was applied. The slices were deparaffinized twice in 100% xylene for 10 min; hydrated with 90%, 85%, 80%, 70%, 60%, and 50% alcohol for 10 min; and washed with distilled water. The 10-mM citrate buffer (pH 6.0) was heated at 750 W for 5 min in a microwave. The slides were then heated twice for 5 min to expose antigens and left at room temperature for 20 min to gradually lower the temperature. Subsequently, the slides were washed with Tris-buffered saline (TBS, 50 mM, pH 7.4), treated with 0.3% hydrogen peroxide for 15 min, and washed thrice with Tris-buffered solution. To block non-specific antigens in the tissues, the slides were treated with normal horse serum for 30 min. The primary antibody that was used was rabbit anti-human c-erbB-2 oncoprotein (DAKO, Denmark, 1:100), and the slides were incubated overnight in a chamber in a 4 °C bath and washed three times with TBS. Subsequently, the slides were treated with a secondary antibody (Vector Elite kit, Vector Laboratories, USA) for 30 min and then reacted with avidin-biotin conjugate (ABC) reagent at room temperature for 45 min. After washing the specimens with TBS and Tris-HCl (pH 7.6) buffered solution, the slides were treated with diaminobenzidine tetrachloride (DAB, Sigma Chemicals, USA) for 2–3 min, washed with distilled water, and stained with 10% Mayer’s hematoxylin.

### Evaluation and classification of IHC

IHC was assessed according to the Hoffmann criteria [27]. Specimens with no staining or staining in less than 10% of all tumor cells were classified as 0, those with faint or hardly noticeable staining in 10% or more of the tumor cells were classified as 1+, those with mild to moderate staining in 10% or more of the tumor cells were classified as 2+, and those with moderate to strong staining in 10% or more of the tumor cells were classified as 3+ and defined as overexpression.

This study classified patients with HER2 0 and 1+ specimens into group 1 (445 individuals), those with HER2 2+ specimens into group 2 (171 individuals), and those with HER2 3+ specimens into group 3 (166 individuals).

### Statistical analysis

Statistical analyses were performed using SPSS (version 23, SPSS, Chicago, IL, USA). Independent two-sample *t* tests and Mann-Whitney tests were used for continuous data, and the chi-square test was used for categorical data. Survival curves were analyzed using the Kaplan-Meier survival analysis, and the log-rank test was used to compare survival rates in each group. In all cases, *p* values < 0.05 were considered as statistically significant.

## Results

### Clinical information of the study participants

The mean patient age was 61.9 (26–92) years, and the male-to-female ratio was 1.93:1. There were 630 patients who underwent curative sub-total gastrectomy (80.5%) and 152 who underwent curative total gastrectomy (19.5%). In the histologic differentiation of gastric adenocarcinoma, 422 patients had differentiated gastric adenocarcinomas (53.9%), including those with well- and moderately differentiated gastric adenocarcinomas. There were also 360 patients with undifferentiated cancers (40.2%), and this cohort included patients with poorly differentiated gastric adenocarcinoma and signet ring cell carcinoma. According to the Lauren classification, we observed 465 intestinal-type cases (59.5%), 314 diffuse-type (40.2%), and 3 mixed-type (0.3%). In terms of T stage, there were 511 T1 cases (65.3%), 79 T2 cases (10.1%), 121 T3 cases (15.5%), and 71 T4 cases (9.1%). In terms of N stage, there were 558 N0 cases (71.4%), 81 N1 cases (10.4%), 60 N2 cases (7.7%), and 83 N3 cases (10.5%). Thus, there were a total of 224 patients with lymph node metastases (28.6%). In terms of TNM stages, there were 524 stage I cases (67.0%), 139 stage II cases (17.8%), and 119 stage III cases (15.2%). There were 180 patients with lymphatic invasion (23.0%), 78 with vascular invasion (10.0%), and 167 with perineural invasion (21.4%), out of 782 patients (Table 1).

**Table 1:** Association of HER2 receptor expression with clinicopathologic factors (782 patients)

	HER2 0/1+	HER2 2+	HER2 3+	*p* value
Age				0.123
≥ 65	186	65	81	
< 65	259	106	85	
Sex				0.013
Male	279	114	122	
Female	166	57	44	
Tumor location				0.953
Upper	64	29	24	
Middle	163	57	59	
Lower	218	85	83	
Tumor size				0.228
≥ 60 mm	48	18	12	
< 60 mm	397	153	154	
Tumor differentiation				< 0.001
Differentiation	210	97	115	
Undifferentiation	235	74	51	
Laurenerentiationtionre				< 0.001
Intestinal type	232	111	122	
Diffuse type	210	60	44	
Mixed type	3	0	0	
T stage				0.022
1	280	109	122	
2	44	24	11	
3	74	26	21	
4	47	12	12	
N stage				0.877
0	318	126	114	
1	43	21	17	
2	34	8	18	
3	50	16	17	
TNM stage				0.202
I	288	122	114	
II	83	27	29	
III	74	22	23	
Lymphatic invasion				0.357
Positive	106	41	33	
Negative	339	130	133	
Vascular invasion				0.476
Positive	40	21	17	
Negative	405	150	149	
Perineural invasion				0.077
Positive	103	37	27	
Negative	342	134	139	

Of the patients who underwent curative gastrectomy, 690 (88.2%) survived and the 5-year overall survival rate was 83.0%.

### HER2 expression level in IHC

There were 358 HER2 0 expression cases (45.8%), 87 HER2 1+ expression cases (11.1%), 171 HER2 2+ expression cases (21.9%), and 166 HER2 3+ expression cases (21.2%) according to the IHC evaluation.

### HER2 expression level and its relationship with clinicopathologic factors (Table 2)

HER2 overexpression was significantly high in males (*p* = 0.013), and HER2 expression was significantly more frequent in patients with differentiated gastric adenocarcinoma (well- and moderately differentiated adenocarcinomas) than in those with undifferentiated gastric adenocarcinoma (poorly differentiated adenocarcinoma and signet ring cell carcinoma) (*p* < 0.001). According to the Lauren classification, HER2 expression was significantly more frequent in intestinal-type adenocarcinoma than in diffuse-type adenocarcinoma (*p* < 0.001). With respect to the T stage, the lower the pathologic stage, the greater was the HER2 expression level and this was statistically significant (*p* = 0.022). HER2 expression level did not have a statistically significant correlation with age (*p* = 0.123), cancer location (*p* = 0.953), cancer size (*p* = 0.228), N stage (*p* = 0.877), TNM stage (*p* = 0.202), lymphatic invasion (*p* = 0.357), vascular invasion (*p* = 0.476), and perineural invasion (*p* = 0.077).

**Table 2:** Determination of predictive factors for 5-year overall survival by univariate analysis

	Hazard ratio	95% CI		*p* value
Age	1.068	1.045	1.093	<0.001
Sex				
Male	1.000			0.474
Female	0.833	0.504	1.375	
Tumor location				
Lower	1.000			0.181
Middle	0.555	0.298	1.036	
Upper	0.687	0.367	1.287	
Tumor size				
< 60 mm	1.000			<0.001
≥ 60 mm	6.029	3.707	9.806	
Tumor differentiation				
Differentiation	1.000			0.010
Undifferentiation	1.869	1.163	3.002	
Laurenereclassification				
Intestinal type	1.000			0.026
Diffuse type	1.837	1.154	2.924	
Mixed type	0.000	0.000		
T stage				
1	1.000			<0.001
2	1.552	0.528	4.562	
3	4.666	2.469	8.821	
4	14.343	8.057	25.530	
N stage				
0	1.000			<0.001
1	1.622	0.665	3.956	
2	2.740	1.235	6.080	
3	10.640	6.212	17.612	
TNM stage				
I	1.000			<0.001
II	2.660	1.303	5.429	
III	11.783	6.751	20.567	
Lymphatic invasion				
Negative	1.000			<0.001
Positive	3.291	2.073	5.225	
Vascular invasion				
Negative	1.000			0.001
Positive	2.541	1.436	4.496	
Perineural invasion				
Negative	1.000			<0.001
Positive	6.785	4.219	10.912	
HER 2 expression				
0/1+	1.000			0.776
2+	0.880	0.526	1.473	
3+	0.844	0.499	1.427	

### Relationship between HER2 expression level and survival rate (Figs. 1, 2, 3 and 4)

According to the HER2 expression level, 445 cases belonged to group 1, 171 cases belonged to group 2, and 166 cases belonged to group 3. The overall 5-year survival rates were 81.9%, 83.7%, and 86.1%, respectively. HER2 expression level and overall 5-year survival rate did not have a statistically significant correlation (*p* = 0.775). The overall 5-year survival rates according to the TNM stage were 92.1%, 88.0%, and 94.2% in groups 1, 2, and 3 respectively for stage I; 83.2%, 84.1%, and 91.7% respectively for stage II; and 36.1%, 62.1%, and 42.2% respectively for stage III. There were no statistically significant correlations between HER2 expression levels and overall 5-year survival rates according to the TNM stage (*p* = 0.756 in stage I, *p* = 0.571 in stage II, and *p* = 704 in stage III).

**Fig. 1:**
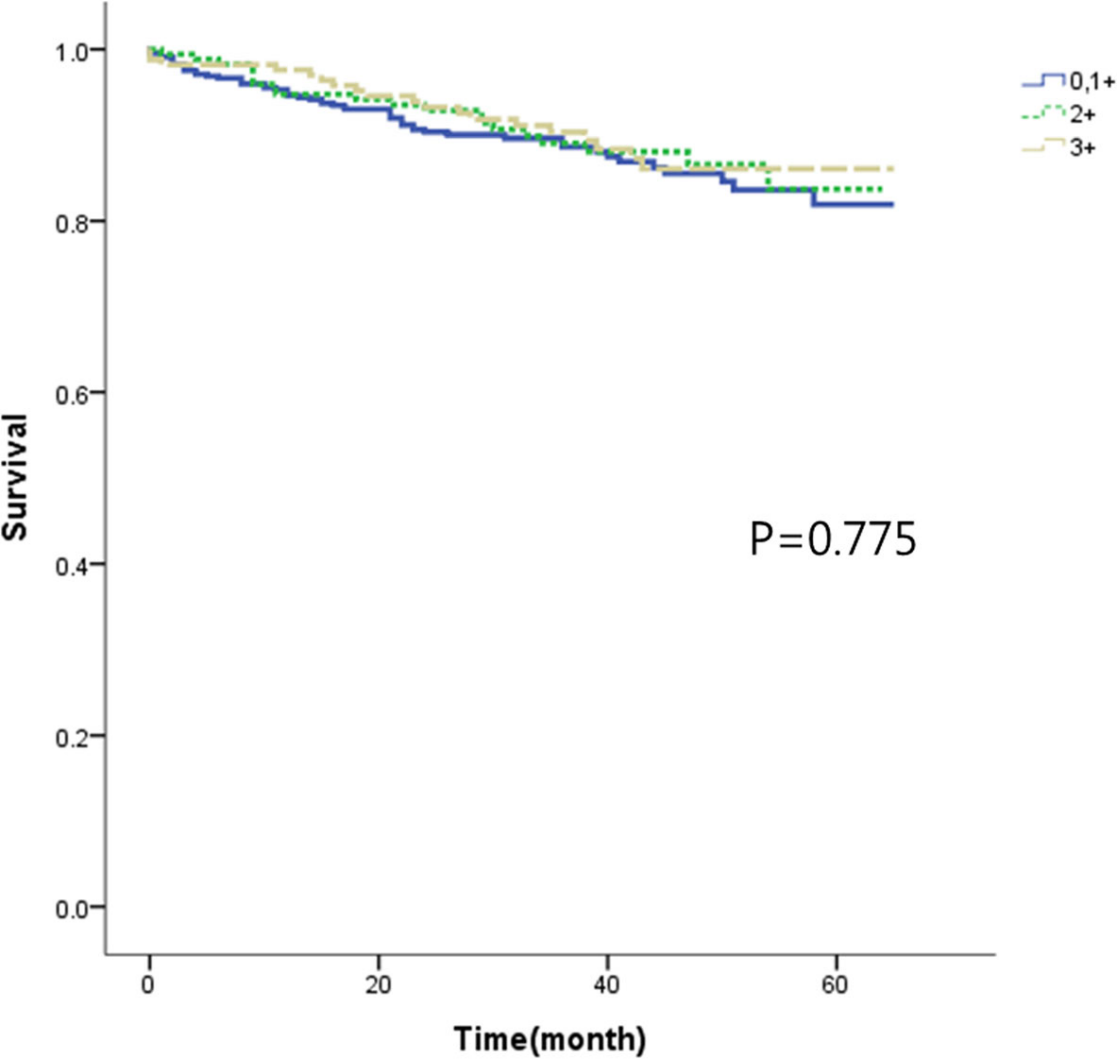
5-year overall survival according to the degree of HER2 receptor in 782 patients with gastric adenocarcinoma

**Fig. 2:**
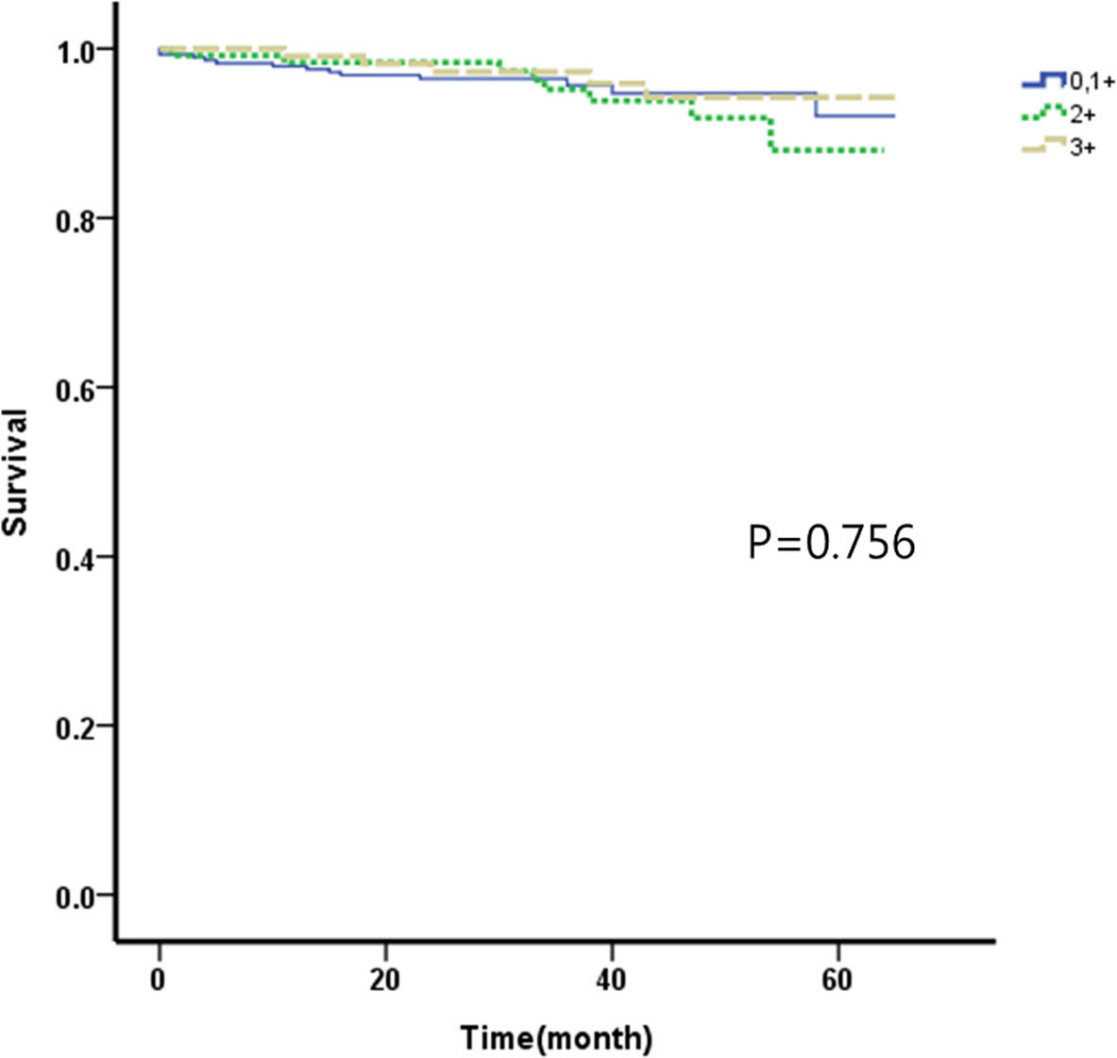
5-year overall survival according to the degree of HER2 receptor in patients with stage I gastric adenocarcinoma

**Fig. 3:**
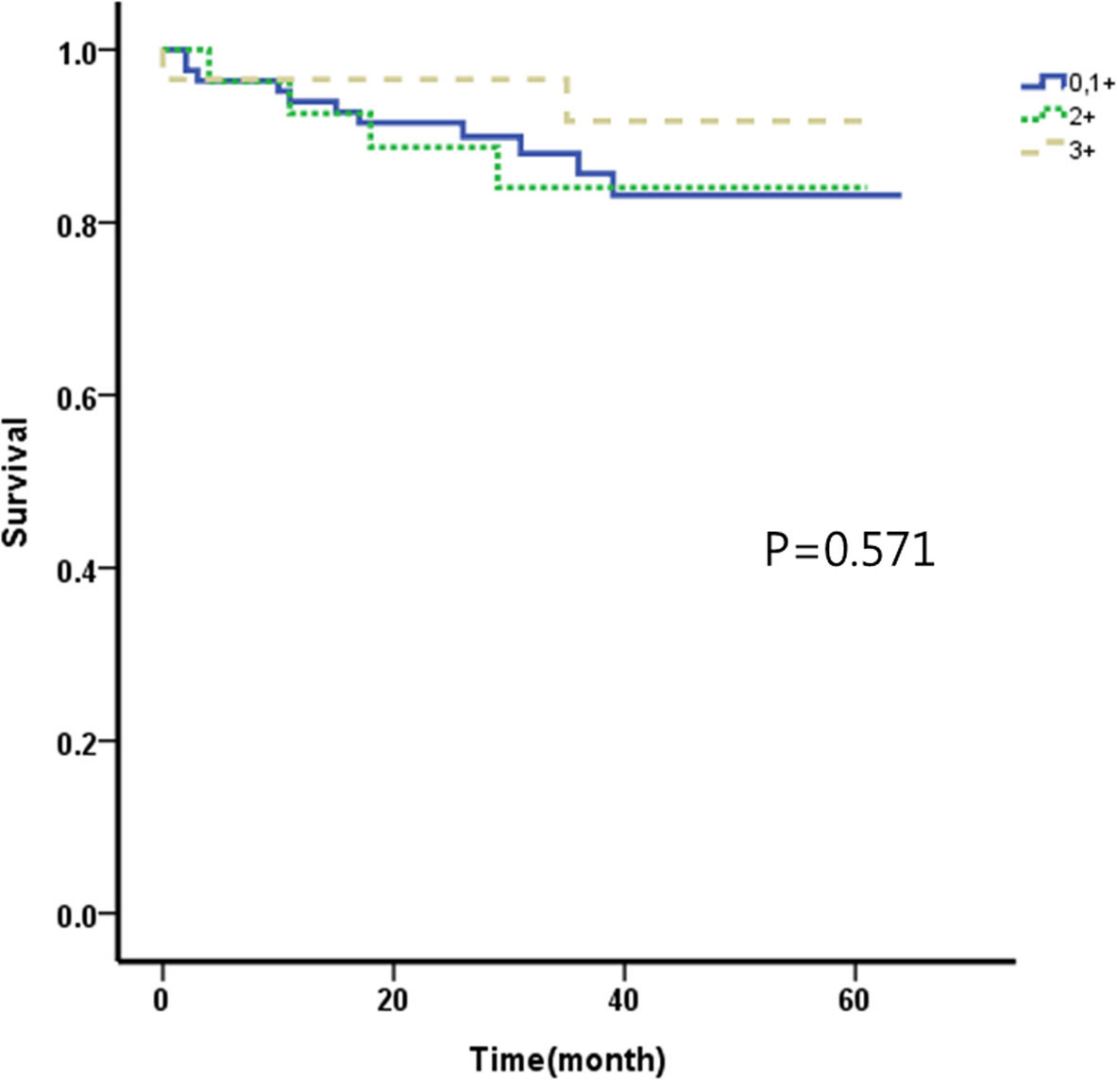
5-year overall survival according to the degree of HER2 receptor in patients with stage II gastric adenocarcinoma

**Fig. 4:**
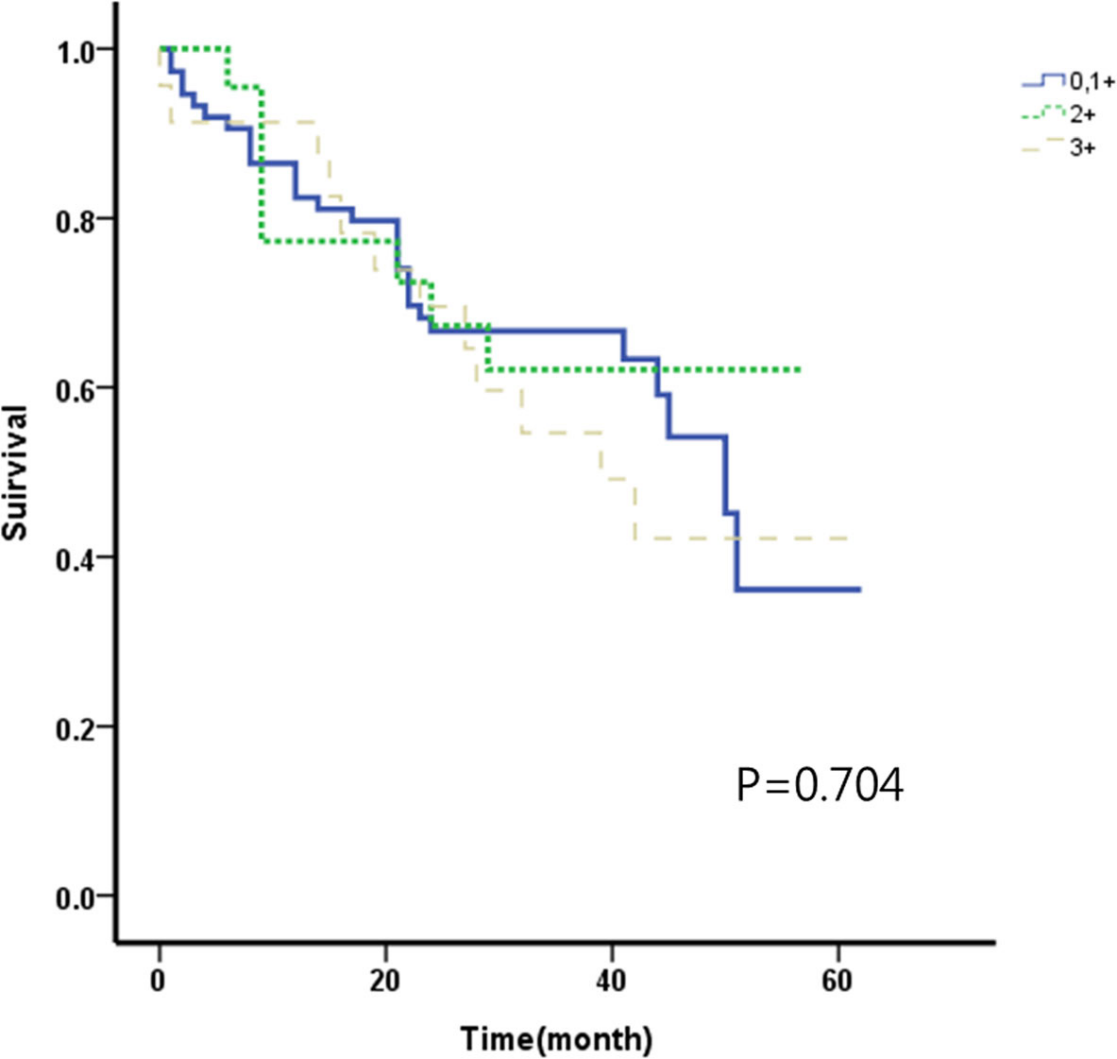
5-year overall survival according to the degree of HER2 receptor in patients with stage III gastric adenocarcinoma

### Univariate and multivariate analyses of survival rates according to HER2 expression level (Tables 3 and 4)

In the univariate analysis, the clinicopathologic factors that affected the overall 5-year survival rates included age (*p* < 0.001), cancer size (*p* < 0.001), histologic differentiation (*p* = 0.010), Lauren classification (*p* = 0.026), T stage (*p* < 0.001), N stage (*p* < 0.001), TNM stage (*p* < 0.001), lymphatic invasion (*p* < 0.001), vascular invasion (*p* = 0.001), and perineural invasion (*p* < 0.001). The clinicopathologic factors that affected the overall 5-year survival rate in the multivariate analysis included age (*p* < 0.001), cancer size (*p* = 0.028), N stages (*p* < 0.001), and perineural invasion (*p* < 0.001). The HER2 expression level was not significantly associated with 5-year survival rate (*p* = 0.776).

**Table 3:** Determination of predictive factors for 5-year overall survival by multivariate analysis

	Hazard ratio	95% CI		*p* value
Age	1.076	1.049	1.104	< 0.001
Tumor size				
< 60 mm	1.000			0.028
≥ 60 mm	1.833	1.067	3.147	
Tumor differentiation				
Differentiation	1.000			0.299
Undifferentiation	1.538	0.682	3.466	
Laurenerentiationtionia				
Intestinal type	1.000			0.555
Diffuse type	0.779	0.340	1.784	
Mixed type	0.000	0.000		
T stage				
1	1.000			0.408
2	0.868	0.242	3.118	
3	1.068	0.228	5.009	
4	1.890	0.344	10.398	
N stage				
0	1.000			< 0.001
1	0.955	0.374	2.442	
2	1.403	0.596	3.301	
3	3.766	1.858	7.637	
TNM stage				
I	1.000			0.699
II	1.940	0.407	9.260	
III	2.875	0.222	37.161	
Lymphatic invasion				
Negative	1.000			0.215
Positive	0.670	0.356	1.262	
Vascular invasion				
Negative	1.000			0.832
Positive	0.931	0.483	1.796	
Perineural invasion				
Negative	1.000			< 0.001
Positive	3.387	1.831	6.267	

## Discussion

In our study, HER2 overexpression was found in 166 of 782 patients (21.2%) who underwent curative gastrectomy for gastric adenocarcinoma. HER2 overexpression was predominantly observed in males, well- and moderately differentiated type according to the histologic differentiation, the intestinal-type according to Lauren classification, and low T stage. There was no correlation between HER2 expression level and overall 5-year survival rate, regardless of the classification method. The clinicopathologic factors that affected overall 5-year survival rate in the univariate analysis were age, cancer size, histologic differentiation, Lauren classification, T stage, N stage, TNM stage, lymphatic invasion, vascular invasion, and perineural invasion. In the multivariate analysis, the important clinicopathologic factors were age, cancer size, N stage, and perineural invasion.

In other studies, HER2 overexpression was defined as HER2 3+ in IHC and as HER2 2+ in IHC and specimens with amplified HER chromosome levels based on fluorescence in situ hybridization (FISH) or silver-enhanced in situ hybridization (SISH). HER2 overexpression rates ranged from 6 to 35% [8–12, 15–18, 20, 22, 28, 29]. IHC and FISH/SISH showed a concordance rate of HER2 expression to be 88.5–93.5% [11, 18, 25, 30, 31]. The rates of specimens with HER2 2+ in IHC and amplified HER2 gene levels based on FISH (or SISH) were 4% [11], 7.7% [8], and 20% [9], respectively. In this study, FISH was not performed in the HER2 2+ IHC specimens due to the high cost of implementing FISH. Based on the high concordance of HER2 expression level in IHC and FISH/SISH and the low HER2 gene amplification rate in FISH/SISH and HER2 2+ in IHC, we defined only HER2 3+ in IHC as HER2 overexpression. Thus, HER2 3+ in IHC was defined as HER2 overexpression among the 782 patients who underwent curative gastrectomy for gastric adenocarcinoma and the HER2 overexpression rate was 21.2%. Instead, the patients were divided into overexpression, non-expression, and other groups to analyze whether the survival rate and prognostic factors were related. If FISH was performed on the specimens from the 171 patients with HER2 2+, the HER2 overexpression rate would have been a little higher.

A multicenter study by Seo et al. [23] showed that HER2 overexpression was related to the intestinal type according to the Lauren classification. Kataoka et al. [15] reported that HER2 overexpression was related to males, old age, and intestinal-type adenocarcinoma. Shen et al. [18] claimed that HER2 expression was associated with sex, tumor location, histologic differentiation, and TNM stage. Begnami et al. [19] reported that HER2 overexpression was related to the degree of tumor invasion, lymph node metastasis, and tumor pathologic stage. Kim et al. [20] reported that age, histologic differentiation, lymphovascular invasion, and lymph node metastasis correlated with HER2 overexpression. Kurokawa et al. [22] reported that histologic differentiation, intestinal-type adenocarcinoma, and pathologic stage correlated with HER2 expression, and Kim et al. [28] reported that sex, age, and intestinal-type adenocarcinoma were associated with HER2 expression. In this study, HER2 overexpression in gastric adenocarcinoma was observed in patients with differentiated-type gastric adenocarcinoma (well- and moderately differentiated adenocarcinoma) according to the histologic differentiation and intestinal-type adenocarcinoma according to the Lauren classification. HER2 overexpression was more frequently found in males than in females and in low T stages. However, unlike other studies, this study did not investigate HER2 expression according to age, cancer location, cancer size, N stage, TNM stage, lymphatic invasion, vascular invasion, and perineural invasion. Among T stages, N stages, and TNM stages related to the progression of gastric adenocarcinoma, the HER2 overexpression was only related to the low T stages. Therefore, HER2 expression seems to show a high level of expression in gastric adenocarcinoma which is in the low stages and has good differentiation.

Although some studies reported that HER2 overexpression led to a poor survival rate [13, 14, 19, 22], others demonstrated that HER2 expression level was not related to overall survival rates [12, 15–18]. Terashima et al. [16] reported no relationship between relapse-free survival and HER2 expression level, and Shen et al. [18] reported that HER2 overexpression was not associated with disease-free survival. In this study, there was no correlation between HER2 expression level and overall 5-year survival rate in patients with resectable gastric adenocarcinoma. The HER2 expression level was also independent of the overall 5-year survival rate when classified according to the pathologic stage. Thus, HER2 expression level did not affect survival rates. This was because HER2 overexpression is not closely related to TNM stages, which was strongly related to the survival rate of gastric adenocarcinoma.

There have been various opinions on the relationship between HER2 expression level and prognostic factors. Patients with gastric cancer with HER2 overexpression have been reported to show poor prognosis [10], and HER2 overexpression has been reported to be an independent prognostic factor for patients with gastric cancer [14, 20–22]. On the other hand, some studies have indicated that the HER2 expression level is not related to the prognosis of gastric adenocarcinoma [12, 16, 18]. In this study, HER2 overexpression did not correlate with the clinicopathologic factors that affect overall 5-year survival rates in univariate and multivariate analyses and HER2 overexpression was not an independent prognostic factor.

Unlike other studies that carried out a retrospective IHC analysis on prepared tissue samples to evaluate HER2 expression level, in this study, we performed a prospective IHC analysis on pathologic tissues obtained from patients who underwent curative gastrectomy for gastric adenocarcinoma, to assess HER2 expression level. In other words, fresh adenocarcinoma tissue obtained during curative gastrectomy was made simultaneously with slides for the pathological stage as well as slides for IHC analysis. However, the HER2 expression level was assessed only by IHC analysis, so patients with HER2 2+ in IHC and HER2 gene amplification in FISH were excluded from the HER2 overexpression group. As a result, this study has shown that HER2 was neither an independent prognostic factor nor an informative prognostic biomarker in resectable gastric adenocarcinomas. However, there may be some bias in the relationship between HER2 expression levels and clinicopathologic factors and in the investigation of the impact of these factors on 5-year survival rates and prognostic factors. In contrast, the ToGA study has shown that the use of trastuzumab in unresectable HER2 positive gastric cancer is effective and patients with HER2-positive breast cancer were treated with trastuzumab. Based on the ToGA study, we will have to compare the effects and side effects of S-1 or xelox treatment and trastuzumab treatment as adjuvant therapy in resectable HER2-positive gastric cancer (stage II and III). An alternative adjuvant therapy should be to treat patients having HER2-positive gastric adenocarcinoma with trastuzumab.

## Conclusion

In this study, IHC analysis of gastric adenocarcinoma tissues from patients who underwent curative gastrectomy was performed to investigate HER2 expression level. The HER2 overexpression rate was 21.2% for patients with gastric adenocarcinoma, and HER2 overexpression was predominantly seen in males, well- and moderately differentiated adenocarcinomas, intestinal type according to the Lauren classification, and low T stage. HER2 expression level did not affect the overall 5-year survival rate of patients with gastric adenocarcinoma and was not correlated with independent gastric adenocarcinoma prognostic factors.

## Data Availability

All data generated or analyzed during this study are included in this published article (and its supplementary information files).
